# Vasotocin receptor blockade disrupts maternal care of offspring in a viviparous snake, *Sistrurus miliarius*

**DOI:** 10.1242/bio.022616

**Published:** 2017-01-09

**Authors:** Craig M. Lind, Nikolette K. Birky, Anita M. Porth, Terence M. Farrell

**Affiliations:** Department of Biology, Stetson University, Deland, FL 32723, USA

**Keywords:** Oxytocin, Social behavior, Reptile, Vasopressin, Parental care, Body condition

## Abstract

Parental care is a complex social behavior that is widespread among vertebrates. The neuroendocrine regulation of parent-offspring social behavior has been well-described in mammals, and to a lesser extent, in birds and fish. However, little is known regarding the underlying mechanisms that mediate the expression of care behaviors in squamate reptiles. In mammalian model species and humans, posterior pituitary hormones of the oxytocin and vasopressin families mediate parental care behaviors. To test the hypothesis that the regulatory role of posterior pituitary neuropeptides is conserved in a viviparous squamate reptile, we pharmacologically blocked the vasotocin receptor in post-parturient pigmy rattlesnakes, *Sistrurus miliarius*, and monitored the spatial relationship between mothers and offspring relative to controls. Mothers in the control group demonstrated spatial aggregation with offspring, with mothers having greater post-parturient energy stores aggregating more closely with their offspring. Blockade of vasotocin receptors eliminated evidence of spatial aggregation between mothers and offspring and eliminated the relationship between maternal energetic status and spatial aggregation. Our results are the first to implicate posterior pituitary neuropeptides in the regulation of maternal behavior in a squamate reptile and are consistent with the hypothesis that the neuroendocrine mechanisms underlying social behaviors are broadly conserved among vertebrates.

## Abstract (precis)

**Summary:** In a squamate reptile, post-birth maternal effort is related to maternal energetic status, and vasotocin receptors may play a role in mediating maternal care.

## INTRODUCTION

Parental care of offspring, defined as any post-fertilization behavior that helps to increase offspring fitness, takes many forms and is widespread among vertebrates (Balshine, 2012; Clutton-Brock, 1991; Gross, 2005). Post-birth parental care is ubiquitous in endothermic birds and mammals (Smith et al., 2012a), but also occurs in fish, amphibians, and reptiles (Crump, 1996; Gross and Sargent, 1985; Kupfer et al., 2006; Lang, 1987; Langkilde et al., 2007; Shine, 1988) as well as a variety of invertebrates (for review see Trumbo, 2012). In birds and mammals, parental care in the form of nutritional provisioning is critical to offspring survival. In other groups, care may not constitute any direct transfer of nutrition/energy, but instead consists primarily of offspring or egg defense, or the facilitation of a proper developmental environment (Smith et al., 2012a). In either case, parental care is a complex social behavior observed across taxonomic groups that use the entire spectrum of vertebrate reproductive strategies and is likely adaptive under varying contexts (Alsonzo-alverez and Velando, 2012). Understanding the neuroendocrine pathways which regulate such complex, fitness-related behaviors in diverse taxa will shed light on how social behaviors evolve at a mechanistic level.

The neuroendocrine regulation of parental care has been described in mammals, birds, and fish (Ross and Young, 2009). In most cases, posterior pituitary peptide hormones are critical in mediating both maternal and paternal care (Fernandez-Duque et al., 2009; Pedersen and Prange, 1985). All vertebrates express two families of posterior pituitary peptides, oxytocin (OT)-like and vasopressin (VP)-like, derived from an ancestral duplication of the arginine vasotocin (VT) gene (Acher, 1996; Goodson and Thompson, 2010). In mammals, OT and VP are the primary hypothalamic peptides released at the posterior pituitary. Homologous peptides exist in other vertebrate taxa. Amphibians, birds, and squamate reptiles (including crotaline snakes; Lazari et al., 2006) express the OT-like mesotocin (MT) and the VP-like VT. Bony fish mostly express the OT homologue isotocin and VT (Insel and Young, 2000). The difference between homologues is typically a single amino substitution (Acher, 1996; Moore, 1992; Moore and Lowry, 1998).

Seminal studies conducted over 30 years ago demonstrated that OT promotes maternal care in rats (Pedersen et al., 1982; Pedersen and Prange, 1979; Pedersen and Prange, 1985). It has since been shown experimentally that OT and the OT receptor mediate maternal care in phylogenetically diverse mammals (Francis et al., 2002), including humans (Ross and Young, 2009), and that MT mediates maternal nest attendance and post-hatching care in domestic chickens, *Gallus domesticus* (Chokchaloemwong et al., 2013). Although much of the research on maternal care has focused on the OT family, VP-like peptides and their receptors also mediate a diverse suite of social behaviors including aggression, interspecific interactions, sexual behavior, and maternal care (Bosch and Neumann, 2008; Dunham and Wilczynski, 2014; Fernandez-Duque et al., 2009; Soares et al., 2012). Additionally, VP-like peptides and their receptors mediate the glucocorticoid response to stress (Rivier and Vale, 1983) and are important mediators of social odor recognition (Wacker and Ludwig, 2012). The VP family of peptides is also critical to the maintenance of water balance in all vertebrate groups, however the antidiuretic effects of VP are mediated though a specialized receptor in mammals (Laszlo et al., 1991). Mammals express three forms of VP receptor: VP1a, VP1b, and VP2. The VP1a receptor is widely distributed in the liver, smooth muscle, and brain and is implicated in a variety of social behaviors, including parental care (Acharjee et al., 2004; Donaldson et al., 2010; Morel et al., 1992; Pedersen et al., 1994). The VP2 receptor is distributed along the collecting ducts of the kidney and mediates the well-known antidiuretic effects of VP (Laszlo et al., 1991). The VP1b receptor is distributed in the pituitary and adrenal gland of mammals and possesses a pharmacological binding profile that is markedly different compared to the V1a receptor (Jard et al., 1986; Kruszynski et al., 1980). In non-mammalian vertebrates that express VT, the VT1a receptor's ligand interaction site is strongly conserved and resembles the VP1a receptor (Goodson and Bass, 2001; Goodson and Thompson, 2010; Mahlmann et al., 1994; Mouillac et al., 1995).

Although the importance of posterior pituitary neuropeptides and their receptors in the evolution of social behavior is becoming increasingly clear in birds and mammals (Insel and Young, 2000), almost nothing is known regarding the role of posterior pituitary neuropeptides in the regulation of parent-offspring social behaviors in squamate reptiles. The dearth of knowledge regarding the regulation of parental care behaviors in reptiles likely stems from the long-standing assumption that parental care (other than egg attendance) is not an important component of reptile life histories (Moore and Lindzey, 1992; Shine, 1988). However, parental care is a key component of the life history of most crocodilians (Kushlan, 1973), and several recent empirical studies indicate its importance in certain other groups of squamates (Greene et al., 2002; Hoss et al., 2014; Langkilde et al., 2007; O'Connor and Shine, 2004). Squamate reptiles have evolved a great diversity of reproductive strategies (Shine, 2003; Shine, 2005; Tinkle and Gibbons, 1977), and have long served as models for the evolution of patterns of parental investment such as viviparity and matrotrophy, both of which have evolved independently in many lineages (Blackburn, 1992). Such diversity provides ample opportunity for comparative study of the ultimate and proximate mechanisms that drive the evolution of parental care behaviors in vertebrates.

Of the over 50 described families in the order Squamata, parental care of neonates has only been described in two: the Scincidae (Langkilde et al., 2007; O'Connor and Shine, 2004) and Viperidae (Graves and Duvall, 1995; Greene et al., 2002; Hoss and Clark, 2014). In both families, care of neonates is restricted to viviparous taxa and does not involve nutritional provisioning. Both viviparity and maternal care behaviors have likely evolved independently in each group (Blackburn, 1992; Greene et al., 2002). In the skink, *Egernia saxatilis*, individuals live in ‘nuclear family’ groups, and care reduces the incidence of infanticide by conspecifics (O'Connor and Shine, 2004). Care in viperids consists of offspring attendance and the spatial aggregation of mother and offspring in the period between birth and the first neonatal ecdysis (shed cycle), which occurs days to weeks after birth (Graves and Duvall, 1995; Greene et al., 2002). Additionally, maternal antipredator behaviors are altered in the presence of a litter, suggesting that care in viperids involves defense (Greene et al., 2002; Hoss and Clark, 2014).

To examine, for the first time, the potential importance of posterior pituitary peptides in the regulation of maternal care behavior in a squamate reptile, we pharmacologically blocked the VT1a receptor in a viviparous viperid snake (the pigmy rattlesnake, *Sistrurus miliarius*) known to exhibit maternal attendance and defense of offspring (Greene et al., 2002). We then monitored the spatial relationship between mothers and their offspring compared to control snakes in the time period between birth and neonatal ecdysis to test the hypothesis that posterior pituitary neuropeptides mediate maternal care behaviors in snakes. If posterior pituitary neuropeptides play a role in regulating maternal care, we predicted that blockade of VT1a receptor signaling would disrupt spatial aggregation of mothers with their offspring.

## RESULTS

### Descriptive results

**Fig. 1.**
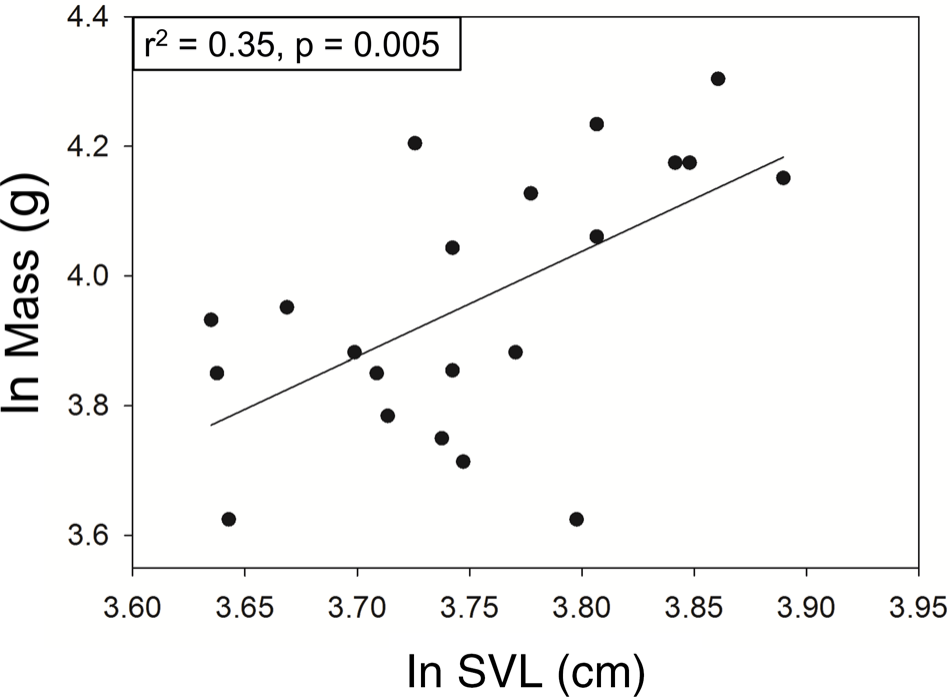
**Scatterplot and linear regression showing the relationship between ln-transformed SVL and ln-transformed postparturient mass.** Residuals of the regression were used to calculate maternal BCI. Linear regression statistics are provided in the top left corner.

Mothers in the control groups and treatment groups were not significantly different in mean snout vent length (SVL), postpartum mass, or body condition index (BCI; *P*>0.05 for all comparisons; Table 1). Mean (±s.e.m.) prepartum holding time in the treatment group was 24.4±3.56 days. Mean holding time in the treatment group was 27.9±2.88 days. Mean holding time was not significantly different between treatment groups (*t*-test: *P*=0.45). Maternal SVL was positively correlated with postpartum mass (Fig. 1).

**Table 1.**
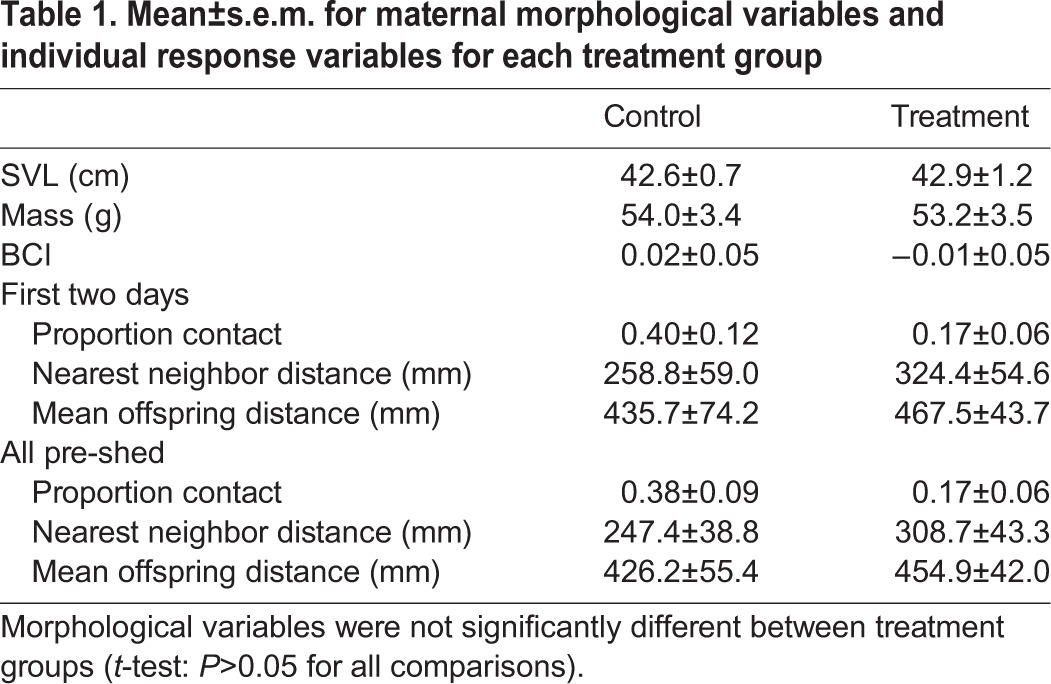
**Mean±s.e.m. for maternal morphological variables and individual response variables for each treatment group**

### Maternal side choice

**Fig. 2.**
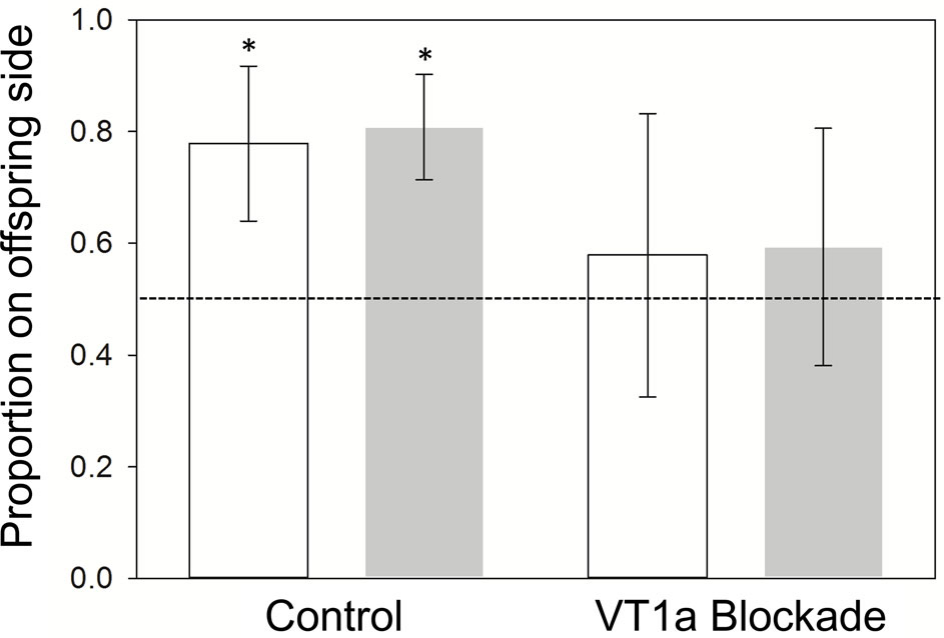
**Means and 95% confidence intervals for the proportion of observations in which the mother was observed on the offspring side of observation enclosures (treatment, *n*=11; control, *n*=10).** Means for the first six observations are in white. All pre-shed data are presented in gray. Confidence intervals indicative of non-random side choice are indicated with a *. The dotted line indicates the expected proportion under random side choice (0.5).

The confidence intervals calculated for the proportion of observations where a mother was observed on the neonate side of the observation arena indicated non-random side choice in the control group, both over the first six observations (CI=0.64–0.83; Fig. 2), and when calculated for all pre-shed observations (CI=0.71–0.90; Fig. 2). Confidence intervals calculated within the VT1a blocked group did not indicate non-random side choice in either the first six observations (CI=0.33–0.83; Fig. 2), or in all pre-shed observations (CI=0.38–0.81; Fig. 2). There was no significant effect of treatment, body condition, or their interaction on the proportion of observations where mothers were on the neonate side of observation arenas (*P*>0.1 for all factors).

### Mother-offspring spatial relationships

**Fig. 3.**
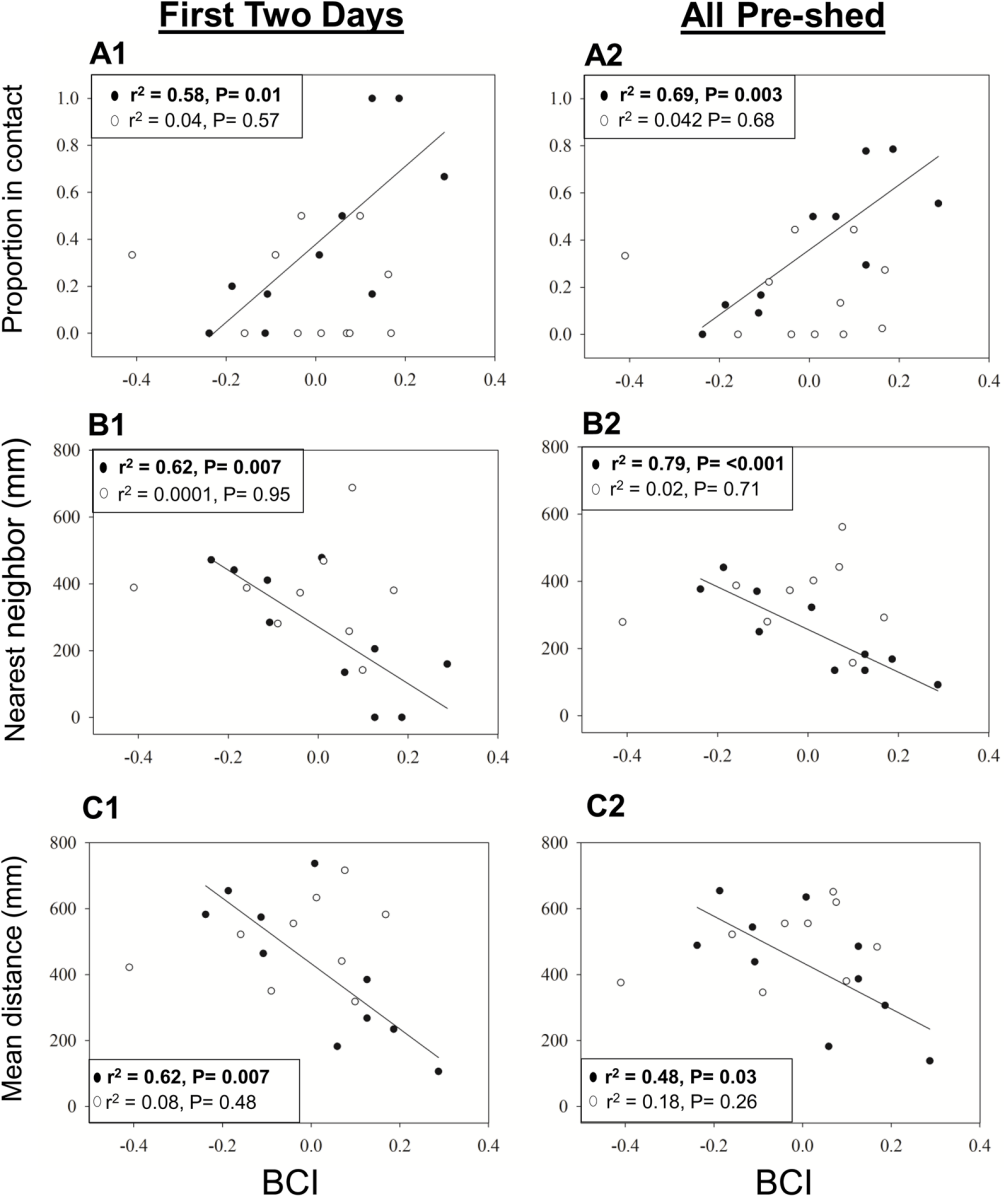
**The relationship between BCI and the three spatial response variables in (1) the first two days of observation and (2) all pre-shed observations.** Unfilled circles represent treatment snakes (AVT blockade). Filled circles represent control snakes. (A1) and (A2) show the proportion of observations in which mothers were observed in contact with at least one offspring (Treatment, *n*=11; Control, *n*=10). (B1) and (B2) show the average mother nearest neighbor distances for each individual. (C1) and (C2) show the mean offspring distance for each individual (Treatment, *n*=9; Control, *n*=10). *P* values and r^2^ for linear relationships between BCI and response variables within each treatment group are indicated on each graph. Linear trend lines are shown for significant linear regressions only.

Analysis of each spatial response was variable for the observations made in the two days after treatment revealed a significant main effect of BCI (*F*_1,17_=4.77, *P*=0.04), a non-significant main effect of treatment (*F*_1,17_=3.93, *P*=0.06), and a significant BCI by treatment interaction effect on proportion contact (*F*_1,17_=8.60, *P*=0.01). When the effect of BCI was analyzed within each treatment group, there was a strong positive relationship between maternal BCI and proportion contact in the control group and not the treatment group (Fig. 3A1). The analysis including all observations before the neonatal ecdysis revealed significant main effects of BCI (*F*_1,17_=5.93, *P*=0.03), treatment (*F*_1,17_=6.35, *P*=0.02) and their interaction (*F*_1,17_=10.00, *P*=0.01) on proportion contact. When each treatment group was analyzed independently by linear regression of BCI on proportion contact, there was a significant positive relationship between BCI and proportion contact in the control group and not the VT1a blocked group (Fig. 3A2). There was a significant treatment by BCI interaction effect on nearest neighbor distance (NND) and mean offspring distance (MOD) in both the analysis of the first two days post treatment (NND: *F*_1,15_=4.33, *P*=0.048; MOD: *F*_1,15_=9.54, *P*=0.01) and in the expanded analysis (NND: *F*_1,15_=7.62, *P*=0.02; MOD: *F*_1,15_=7.97, *P*=0.01). When the effect of body condition was analyzed independently within each treatment group, linear regressions revealed a strong negative relationship between maternal BCI and both NND and MOD in the control groups, but not in the VT1a blocked group (Fig. 3).

## DISCUSSION

The analysis of maternal side choice demonstrated that maternal association with neonates occurred in the control group but did not occur in the VT1a-blocked group (Fig. 1). Although there was no significant effect of treatment in the analysis of side choice, snakes administered Manning compound did not choose to be on the offspring side at a frequency that indicated non-random choice (Fig. 1). This result indicates that spatial association of mothers with offspring occurred in the control group under our experimental conditions and supports the hypothesis that blockade of VT1a receptors disrupts maternal association with offspring in pigmy rattlesnakes.

Analysis of all spatial response variables revealed that the spatial aggregation of a mother with her offspring is strongly related to maternal body condition in control snakes, but not in snakes that received the VT1a antagonist (Fig. 2). Within the control group, mothers with high postparturient energy stores aggregated more closely with their offspring compared to mothers with low postparturient energy stores. The dependence of spatial association on the energetic status of mothers after birth suggests that maternal care behaviors in snakes may come at a cost in terms of time or energy to the mother. As predicted by prior studies, mothers may act selfishly in their own interest and allocate less time and energy toward their current reproductive investment to ensure future fecundity and maximize reproductive success when resources are limited (DeNardo et al., 2012; Schwarzkopf and Andrews, 2012; Trivers, 1974). Blockade of VT1a signaling disrupted the relationship between maternal attendance and body condition, and the effect of VT1a blockade persisted for the duration of the offspring's first shed cycle (up to 5 days after treatment; Fig. 2). This result supports our initial hypothesis that the VT1a receptor is part of the regulatory pathway mediating maternal care in pigmy rattlesnakes.

Our study is the first to experimentally examine potential neuroendocrine mechanisms underlying maternal care behaviors in non-avian reptiles. The effects of VT1a receptor blockade via peripheral injection of Manning compound are similar to those observed in rats, where pup retrieval and maternal nursing behaviors were blocked by central infusion of pituitary peptide receptor antagonists (Pedersen et al., 1994). Peripheral administration of V1a antagonists has also been demonstrated to block social behaviors in diverse vertebrate groups (Propper and Dixon, 1997; Soares et al., 2012). Much of the research concerning blockade of V1a receptors attributes observed effects to signaling by VP-like peptides alone. However, there is some uncertainty as to whether blockade of V1a receptors exclusively disrupts signaling by the VP family. Recent research has demonstrated that there is ‘cross-talk’ between the VP and OT systems in mammals, and that some of the effects of OT on maternal behavior in hamsters are mediated by central VP1a receptors (Song, 2016). Additionally, Manning compound has some affinity for the mammalian OT receptor. It is unclear whether Manning compound antagonizes the MT receptor of non-mammalian vertebrates, or whether cross-talk exists between the MT and VT systems. We can therefore not say with certainty that the observed effects of VT1a blockade were solely due to disruption of VT signaling. Our results highlight the potential importance of the VT1a receptor and the MT/VT system in mediating maternal behavior in snakes.

The strong positive relationship between maternal energetic status and maternal association with offspring observed in our system provides a model for investigation of the interplay between stress/energy signaling, hypothalamic peptides, and maternal investment in offspring. The ultimate (evolutionary) and proximate (ecophysiological) mechanisms underlying the decision to invest a given amount of time and energy in offspring have been the target of thousands of empirical and theoretical studies. Care behaviors may represent a significant portion of an individual's time-energy budget, and are subject to tradeoffs between current and future fecundity. Maternal energy reserves alter maternal investment in a variety of vertebrate species, including snakes (DeNardo et al., 2012; Fairbanks and McGuire, 1995; Markman et al., 2002; Smith and Wootton, 1995), and hormones associated with fat stores, such as leptin, alter post birth investment in mammals (French et al., 2009). Additionally, glucocorticoids associated with energy limitation, stress, and the periods just before and after parturition in reptiles (Hoss et al., 2014; Moore and Jessop, 2003; Schuett et al., 2004; Smith et al., 2012b) may mediate the level of maternal investment in offspring (Angelier and Chastel, 2009). Links between VT/OT-like peptides and stress and energetic status have been demonstrated in mammalian models. Vasopressin stimulates the hypothalamic pituitary adrenal axis and increases circulating cortisol in mammals (Axelrod and Reisine, 1984), and central administration of leptin increases circulating vasopressin in rabbits (Matsumura et al., 2000). It is likely that the ‘decision’ to invest in current fecundity through maternal care of free-living offspring is the product of the complex neuroendocrine interaction of multiple regulatory pathways. Research has identified many aspects of this regulatory pathway in model systems under laboratory conditions. However, if comparative approaches are to be used to understand the evolutionary and physiological basis of parental care behavior, much work needs to be done to elucidate mechanistic regulatory pathways and their interaction with the environment in lineages representing diverse phylogenetic and environmental contexts.

Lepidosaurs (including squamates) diverged from other extant amniotes (e.g. the archosaurs) over 250 million years ago (Shedlock and Edwards, 2009), and have since radiated into a reproductively diverse vertebrate group (Vitt, 1992). Such diversity makes squamates an ideal model for investigating the ultimate and proximate forces underlying reproductive tactics, including patterns of maternal investment in offspring (Blackburn, 2006). Pigmy rattlesnakes are viviparous, primarily lecithotrophic (although some degree of matritrophy has been demonstrated in other crotalines; Van Dyke and Beaupre, 2012), and fall toward the capital end of the capital-income breeding spectrum (Lourdais et al., 2002). Additionally, crotaline snakes typically feed infrequently compared to most other vertebrates, making them an excellent squamate model for investigations of the effect of energetic status on resource allocation (Beaupre and Duvall, 1998). The interactions among hormones associated with stress and energetic status (e.g. glucocorticoids and leptin), sex steroids (e.g. testosterone and estradiol), and peptide hormones implicated in maternal care (e.g. OT/VP and prolactin) can be addressed through both descriptive measurement of circulating hormone concentrations and experimental manipulation utilizing crotaline snakes and other reptiles as models. Elucidation of these complex, integrative, neuroendocrine pathways is vital to understanding both the evolution of parent-offspring social behaviors and the mechanistic underpinnings of environmental adjustments to investment in offspring after birth.

In conclusion, this study is the first to implicate the VT1a receptor in the regulation of maternal care behavior in a non-avian reptile. Our work is limited in that it examines the effect of receptor antagonism, and does not identify specific roles for VT or MT in the pathway regulating care behaviors. The lack of understanding regarding the vasopressor and antidiuretic effects of the VT1a receptor in squamate reptiles also prevents the elimination of the possibility for pharmacological side-effects unrelated to neuroendocrine pathways specific to maternal care. However, antagonism of VT1a receptors via peripheral injection of Manning compound has been used to demonstrate both increases and decreases in social behaviors in a variety of taxa (Donaldson et al., 2010; Dunham and Wilczynski, 2014; Goodson et al., 2004; Propper and Dixon, 1997; Soares et al., 2012), and no studies to our knowledge document any antidiuretic or pharmacological side effects. As one of the few squamate groups known to exhibit maternal care of free-living offspring, crotaline snakes provide an excellent model for future research aimed at elucidating the causal neuroendocrine pathways that modulate post-birth maternal investment in reptiles.

## MATERIALS AND METHODS

### Animals and housing

21 pregnant female pigmy rattlesnakes, *Sistrurus miliarius* (Linneaus 1766), were collected from Lake Woodruff National Wildlife Refuge and Lake Monroe Conservation Area in Volusia County, Florida. Collection dates ranged from June 29th to August 3rd, 2015. Snakes were housed at Lake Woodruff National Wildlife Refuge in subdivided 0.9×1.2 m wooden outdoor enclosures until parturition. Holding time in outdoor enclosures ranged from 2–46 days. During pregnancy, snakes were not fed and were provided water *ad libitum*. Because the variance in maternal association with offspring has not been described in squamates, sample size was selected based on what was logistically feasible and has been demonstrated to establish behavioral effects of pituitary peptide receptor blockade in other species (e.g. Goodson et al., 2004).

Enclosures were monitored daily for the presence of litters. On the day following parturition, females and offspring were assigned randomly to one of two sides of an outdoor 0.9×1.2 m observation arena. To allow free movement of mothers and to restrict offspring to one half of the arena, a 0.2 m high wooden barrier separated the arena into equal-area right and left sides. Each side of the arena contained two uniform hide boxes and a small water dish. To minimize any potential effects of the approaching observer, each enclosure was equipped with a ∼1.4×1.2 m observation blind constructed from a single layer of shade cloth. Blinds angled to the center of the arena where a camera was mounted on the wooden frame of the observation blind such that the observer could monitor behavior by video or a photograph while remaining hidden behind the blind.

All snake handling procedures followed Beaupre and Greene (2012). Female SVL was measured in a squeeze box and mass was taken using a Pesola^®^ spring scale. After parturition, a small blood sample (0.2–0.35 ml) was taken from the caudal vein of each female as part of another study. All animal care, handling, and experimental procedures were approved by the Stetson University Institutional Animal Care and Use Committee (IACUC).

### Experimental procedure

To determine the effect of blockade of VT1a receptors on maternal care behaviors, mothers were randomly assigned to control (*n*=10) and treatment (*n*=11) groups and administered the appropriate treatment. Mothers were then introduced to their randomly assigned side of the arena immediately after the addition of neonates to a randomly assigned side of the arena. Mothers received treatments the morning after parturition was confirmed. Blockade of VT1a receptors was achieved by intraperoteneal injection of a 15 μg per 50 μl saline solution of Manning compound [(β-Mercapto-β,β-cyclopenta-methylenepropiony^l^, O-Me-Tyr^2^,Arg^8^)-Vasopressin; Bachem California, Torrance, CA]. Manning compound is a potent VP1a receptor antagonist (Manning and Sawyer, 1989), has little diuretic activity (i.e. does not antagonize the VP2 receptor), and has some affinity for mammalian oxytocin receptors (Manning et al., 2012). As a result of the structural similarities in ligand binding regions between VP1a and VT1a receptors (Goodson and Bass, 2001; Mouillac et al., 1995), Manning compound also antagonizes the VT1a receptors of fish and birds, and does so selectively (Goodson et al., 2004). Based on the only previous behavioral study that examined the effect of AVT blockade in squamates (Dunham and Wilczynski, 2014), the volume of injections was adjusted to a dose of 3 μg g^−1^ postparturient body mass. Control mothers were injected with a saline solution matched to the volume of equally sized treatment snakes.

Behavioral monitoring began 30–60 min after treatment. Each individual was monitored for 10 min three times per day; once between 08:00 and 10:00 h, once between 11:00 and 13:00 h, and again between 14:00 and 16:00 h. At each observation we initially observed the maternal side choice and whether the mother was in physical contact with her offspring, and then completed a 10 min video recording. Daily observations continued until neonatal ecdysis.

### Data collection

Reproductive time-energy allocation in snakes often depends on factors such as size and energetic status (Lind and Beaupre, 2015). Therefore, maternal SVL, mass, and body condition were analyzed as potential covariates in the analysis of the effect of treatment on behavioral responses. BCI was calculated by taking the residual of the linear regression of natural log transformed mass on natural log transformed SVL (Fig. 1), and was used to compare the relative energetic status of postparturient females. At the onset of the experiment, we planned to monitor behavior for the first two days (six observations) after treatment because it was unlikely for neonatal ecdysis (thought to mark the end of the care period based on other studies; Graves and Duvall, 1995; Greene et al., 2002) to occur before 2 days after birth, and the duration of VT1a blockade in a reptile was not known. This time-period allowed mothers time to settle into their new surroundings and resume care behaviors, but was also short enough that even a short-term effect of treatment could be picked up in statistical analyses. We converted the first six observations into four spatial response variables: (1) proportion of observations where the mother was on the neonate side of the arena, (2) the proportion of observations in which a mother was in physical contact with at least one offspring (Proportion contact), (3) mother NND, and (4) MOD to mother. To examine the duration of the effects of VT1a blockade, an additional analysis was performed on all observations made before neonatal ecdysis (all pre-shed; up to 5 days or 15 observations post-treatment for some individuals).

The spatial relationship of mothers relative to offspring was quantified from analysis of videos recorded at each observation. A still shot was taken from the first clearly-focused segment of video to document the position of each offspring relative the mother. ImageJ digital imaging software (Schneider et al., 2012) was used to calculate the distance of each offspring from its mother. Measurements were taken by observers who were blind to snake treatment. Nearest neighbor distance was calculated as the distance between the mother and her nearest offspring. Mean offspring distance was calculated as the sum of the distance of each individual offspring from their mother divided by the number of offspring measured.

### Statistical analysis

All analyses were conducted in JMP^®^, version 11 (SAS institute Inc., Cary, NC, USA). *t*-tests were used to (1) determine whether any morphological differences existed between treatment groups, and (2) determine whether holding time in cages was significantly different between treatment groups. To determine whether mothers were actively choosing to aggregate with offspring, the proportion of observations for which each mother was observed on the offspring side of the arena was quantified and used to calculate the 95% confidence intervals (CIs) for the proportion of observations that mothers were observed on the offspring side within each treatment group. Confidence intervals were compared to the expected value under random side choice (0.5) and were used to establish maternal association.

Of the three covariates analyzed, only BCI had a significant effect. Therefore, data were analyzed by fitting a general linear model examining the fixed effect of hormone treatment varied at two levels, BCI as a continuous covariate, and their interaction on each of the three univariate spatial response variables. All data satisfied the assumptions of parametric statistics (i.e. normality and homoscedasticity). In two snakes, inclement weather resulted in fewer than four interpretable video observations. Data from these mothers were removed from the analysis of distance variables, resulting in a sample size of 19. Visual observations were recorded on these occasions and data on proportions (contact and enclosure side) include all 21 mothers.
